# A retrospective study of secondary bacteraemia in hospitalised adults with community acquired non-typhoidal Salmonella gastroenteritis

**DOI:** 10.1186/1471-2334-13-107

**Published:** 2013-02-27

**Authors:** Christopher M Parry, Sherine Thomas, Esther J Aspinall, Richard PD Cooke, Stephen J Rogerson, Anthony D Harries, Nicholas J Beeching

**Affiliations:** 1 Tropical and Infectious Disease Unit, Royal Liverpool University Hospital, Liverpool, UK; 2 School of Clinical Infection, Immunology and Medical Microbiology, Institute of Infection and Global Health, University of Liverpool, Liverpool, UK; 3 Centre for Tropical Medicine, Nuffield Department of Clinical Medicine, University of Oxford, Oxford, UK; 4 Mahidol-Oxford Tropical Medicine Research Unit, Faculty of Tropical Medicine, Mahidol University, Bangkok, Thailand; 5 Department of Medical Microbiology, University Hospital Aintree, Aintree, UK; 6 Health Protection Scotland, Glasgow, Scotland, UK; 7 Department of Medicine (RMH), University of Melbourne, Parkville, VIC 3010, Australia; 8 London School of Hygiene and Tropical Medicine, London, UK; 9 International Union Against Tuberculosis and Lung Disease, Paris, France; 10 Clinical Group, Liverpool School of Tropical Medicine, Liverpool, UK

**Keywords:** Salmonella, Bacteraemia, Gastroenteritis, Adults, Complications

## Abstract

**Background:**

The clinical significance of bacteraemia secondary to non-typhoidal *Salmonella* (NTS) gastroenteritis in hospitalised adults is uncertain.

**Methods:**

Adults admitted to a hospital in Liverpool, UK, with NTS gastroenteritis were identified using hospital discharge data and laboratory records. Patients with known HIV infection were excluded. Risk factors for a complicated or fatal course were determined.

**Results:**

Between 1982 and 2006 inclusive, 633 adults were identified. Serovars causing infection included Enteritidis (46.6%), Typhimurium (27.6%) and Virchow (4.9%). A blood culture was taken in 364 (57.5%) patients who were generally sicker than those who were not cultured. Bacteraemia was detected in 63 (17.3%) patients who had blood cultures taken (63/633 (10.0%) of all patients). Bacteraemia was more common in those aged ≥ 65 years (p < 0.001) and in those aged < 65 years who had an underlying chronic disease. A complicated course occurred in 91 (25.0%) patients who had had a blood culture taken (148/633 (23.4%) of all patients). Independent factors associated with a complicated or fatal course among the patients investigated with a blood culture were bacteraemia (Adjusted Odds Ratio 5.34, 95% CI 2.86–9.95); new onset confusion or coma (AOR 4.80, 95% CI 1.91–12.07); prolonged symptoms prior to admission (AOR 2.48, 95% CI 1.44–4.27); dehydration (AOR1.90, 95% CI 1.07–3.38); and absence of fever (AOR 0.56, 95% CI 0.32–0.95). The 30 day attributable case fatality for all patients was 1.5%.

**Conclusions:**

In this study secondary bacteraemia, as well as other clinical factors, was independently associated with a complicated or fatal course in non-HIV infected adults admitted to hospital with NTS gastroenteritis.

## Background

Non-typhoidal *Salmonella* (NTS) organisms are a common cause of bacterial food poisoning. The burden is significant, with an estimated 93.8 million cases worldwide and 155,000 deaths each year [1]. Clinical manifestations range from subclinical infection to severe life-threatening disease. Although NTS infections typically present with diarrhoea that is self-limiting without the need for antibiotic therapy they may also be complicated by extraintestinal infection, including bacteraemia that is primary (in the absence of recent or current including mild and clinically ignored gastroenteritis), or secondary (secondary to gastroenteritis) [2,3]. Primary bacteraemia is linked with a high mortality and is associated with underlying malignancy and immunosuppression, and infection with particular serovars such as Choleraesuis [2-4]. The incidence and significance of secondary bacteraemia is less clear. Many studies mix primary and secondary cases together and patients with apparently uncomplicated gastroenteritis are usually not investigated with a blood culture [5-15]. The extent to which secondary bacteraemia contributes to poor outcomes in NTS gastroenteritis is uncertain.

We report a retrospective study of non-HIV infected adults admitted to hospital with NTS gastroenteritis in Liverpool, UK during a twenty five year period. The purpose of this study was to determine the clinical significance of secondary bacteraemia and in particular if it is important in contributing to adverse outcome in this group of patients.

## Methods

### Study design

This was a retrospective case note review of all adults with NTS gastroenteritis admitted to a single centre. Ethical approval for the study was given by the Fazakerley and Walton Hospitals Research Ethics Committee. All clinical samples taken were part of standard care.

### Data source and collection

Case notes were reviewed for all adults admitted to University Hospital Aintree (previously Fazakerly and Walton Hospitals) Liverpool, with infections due to *Salmonella* species between 1^st^ January 1982 and 31^st^ December 2006 inclusive. The hospital has over 800 beds, including a Regional Infectious Disease Unit (until 2002) where the majority of these patients were managed. Cases were identified from: microbiology laboratory records, Hospital Activity Analysis computer records of in-patient stays and discharge diagnoses, and the manual and computerised patient registers of the Infectious Disease Unit. Clinical and laboratory data for each patient were recorded on a standardised case report form.

Patients > 15 years old, with a diagnosis of acute NTS gastroenteritis were included. Gastroenteritis was considered acute if the onset was ≤ 14 days prior to admission. Known chronic faecal carriers, faecal carriers without gastroenteritis on admission, those with a primary extra-intestinal infection, those with healthcare associated infection (infection onset > 48 hours after admission), patients with non-acute infection (ill for more than 14 days prior to admission), and patients with known HIV/AIDS were excluded from the analysis.

### Bacteriology

Blood cultures were performed using the BACTEC® system (Becton Dickinson, UK). Faecal samples were cultured on routine bacteriology media used for enteric pathogens (all media, Oxoid, Basingstoke, UK). The isolated organisms were identified by standard biochemical tests including the API system (bioMérieux, UK) and serotyping with specific antisera (BioRad, UK). The identification and serovar of isolates was confirmed by the Laboratory for Gastrointestinal Pathogens, Health Protection Agency, Colindale and phage typing was performed for serovar Enteritidis and Typhimurium. Antibiotic susceptibility tests were not performed on *Salmonella* isolates from faeces. Invasive isolates were tested by disk susceptibility to ampicillin, co-trimoxazole and, from 1987 onwards, ciprofloxacin. Nalidixic acid testing was not done and isolates were not retrievable for additional testing.

### Definitions

The following definitions were used: elderly - ≥ 65 years at admission; chronic underlying disease – the presence of hypertension, ischaemic heart disease, valvular heart disease, congestive heart failure, peripheral vascular disease, cerebrovascular disease, chronic pulmonary disease, renal impairment, chronic liver disease, inflammatory bowel disease, connective tissue disease, diabetes mellitus, dementia or malignancy; vascular and valvular heart disease – the presence of hypertension, ischaemic heart disease, valvular heart disease, congestive cardiac failure, cerebrovascular disease, peripheral vascular disease or diabetes mellitus; hypochlorhydria – documented past gastric surgery, pernicious anaemia, or current H_2_ antagonist or proton pump inhibitor therapy (H_2_A/PPI); fever – temperature > 38°C during the first 24 hours of admission; tachycardia – pulse >90/minute; hypotension – systolic blood pressure <90 mmHg; dehydration – loss of normal skin turgor or dry mucous membranes; mental impairment – an abnormal state of consciousness; anaemia - haemoglobin <11 g/dl in females and <13 g/dl in males; leucocytosis –white blood cell count >12.0 × 10^9^/L; leucopenia - white blood cell count of <4 × 10^9^/L; elevated urea – urea >7.5 mmol/l; bacteraemia – one or more blood cultures positive for *Salmonella* spp; bacteriuria – one or more urine cultures positive for *Salmonella* spp, plus one or both of urinary symptoms or urine white cell count ≥50 cells/μL.

Secondary bacteraemia was defined by the isolation of *Salmonella* from blood following an episode of gastroenteritis in the previous month associated with the isolation of the same *Salmonella* from faeces and from no other site except faeces. The bacteraemia was defined as community acquired bacteraemia if the onset was in the community or within 48 hours of hospital admission. Salmonella carriage was defined by the isolation of salmonella from faeces on one or more occasions in the absence of any relevant gastrointestinal symptoms. Complicated disease was defined on the basis of clinical features and, in some instances, culture positivity. Complicated disease included: any gastrointestinal complication excluding post infection *Salmonella* faecal carriage; extra-intestinal complications including a vascular event or evidence of vascular infection; clinical evidence of pneumonia; evidence of a urinary tract infection with NTS (urine microscopy evidence of ≥50 leucocytes/μL and ≥ 10^5^/L pure growth of NTS); reactive arthritis or uveitis; the general complications of infection including hypotension; renal impairment (new creatinine >300 mmol/l with urea >7.5 mmol/l); a new onset of acute confusion or coma; an admission duration >14 days; or fatal disease. We considered most patients with NTS gastroenteritis would be admitted to hospital for less than two weeks and that a prolonged admission of more than two weeks was an adverse outcome of the illness. Although there are many reasons why the duration of admission could be prolonged, and difficult to disentangle in a retrospective study, in most instances the prolonged admission was directly related to the initial nfection. The attributable case fatality was the proportion of deaths that occurred in hospital, in the period up to 30 days following admission, and that were considered attributable to the NTS infection.

### Analysis

Non-normally distributed variables were compared using the Mann-Whitney U test. Categorical variables were compared using the Chi squared test, or Fisher’s exact test. A significance level of p <0.05 was used for all analyses. Variables found to be significant on univariable analysis were entered into a backward logistic regression model, and adjusted odds ratios calculated for each variable. Cases with data missing for the variables entered into the logistic regression were excluded from this analysis [16]. All statistical analysis was performed using SPSS for Windows v 18 (SPSS inc, Chicago, USA) and Epi-info software (CDC, Atlanta, USA).

## Results

**Figure 1:**
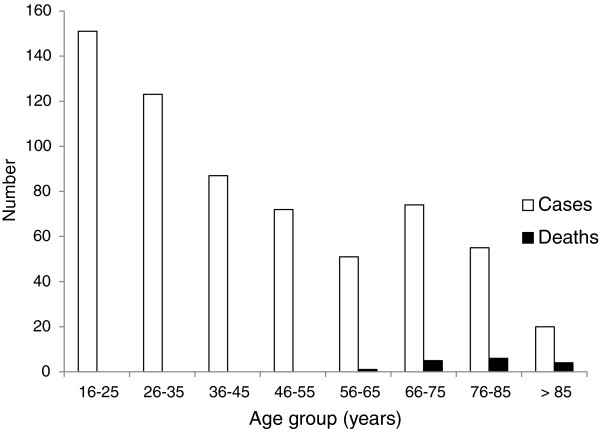
**Number of cases, fatal cases and case fatality rates by age group in 633 adults admitted to a hospital in Liverpool, UK with non-typhoidal *Salmonella *gastroenteritis.**

A total of 754 patients with a *Salmonella* isolated from any site were identified. 121 patients were excluded: 40 with non-typhoidal faecal *Salmonella* carriage; 16 with healthcare associated *Salmonella* infection; 14 patients under 16 years of age; 25 with pre-admission symptoms for longer than 14 days; 12 with serovar Typhi or Paratyphi; 12 with primary extra-intestinal infections; and two with known HIV/AIDS. The remaining 633 patients were included in the analysis. The characteristics of the 633 patients are in Table 1. There were 202 (31.9%) patients with documented chronic disease principally vascular and valvular heart disease (99); hypochlorhydria (71); chronic lung disease (22); malignancy (15) and connective tissue disease (12). Figure 1 shows the age distribution of cases.

**Table 1:** **Characteristics of 633 adults admitted to hospital with non-typhoidal *Salmonella *gastroenteritis in Liverpool between 1982 and 2006 and of the 364 patients who had a blood culture taken**

	**All patients**	**Patients with blood culture taken**	**Patients with no blood culture taken**	**p**^**1**^	**Positive blood culture**	**Negative blood culture**	**p**^**2**^
Number	633	364	269		63	301	
Median age (IQR, range) years	40 (26–64,16-98)	39 (26–61,16-91)	42 (26–68,19-98)	0.083	62 (36–75,16-91)	35 (25–53,16-90)	<0.001
Age ≥ 65 years	150 (23.7)	76 (20.9)	74 (27.5)	0.059	27 (42.9)	49 (16.3)	<0.001
Male gender	300 (47.4)	171 (47.0)	129 (48.0)	0.810	31 (49.2)	140 (46.5)	0.782
Current smoker	216 (34.1)	113 (31.0)	103 (38.3)	0.573	20 (31.7)	93 (30.9)	0.895
Current alcohol user	369 (58.3)	224 (61.5)	145 (53.9)	0.541	35 (55.6)	189 (62.8)	0.283
Recent travel history	79 (12.5)	49 (13.5)	30 (11.2)	0.398	7 (11.1)	42 (14.0)	0.686
Chronic disease	202 (31.9)	113 (31.0)	89 (33.1)	0.605	29 (46.0)	84 (27.9)	0.007
Hypochlorhydria	71 (11.2)	39 (10.7)	32 (11.9)	0.703	13 (20.6)	26 (8.6)	0.012
Recent antibiotic therapy	56 (8.8)	32 (8.8)	24 (8.9)	1.00	2 (3.2)	30 (10.0)	0.091
Duration symptoms > 4 days	255 (40.3)	134 (36.8)	121 (45.0)	0.041	26 (41.3)	108 (35.9)	0.473
Bloody diarrhoea	126 (19.9)	65 (17.9)	61 (22.7)	0.158	16 (9.5)	59 (19.6)	0.070
Abdominal pain	493 (77.9)	284 (78.0)	209 (77.7)	0.923	44 (69.8)	240 (79.7)	0.095
Vomiting	427 (67.5)	265 (72.8)	162 (60.2)	0.001	47 (74.6)	218 (72.4)	0.876
Pyrexia	234 (37.0)	199 (49.5)	54 (20.1)	<0.001	27 (42.9)	153 (50.8)	0.270
Rigors	95 (15.0)	83 (22.8)	12 (4.5)	<0.001	8 (12.7)	75 (24.9)	0.046
Headache	142 (22.4)	108 (29.7)	34 (12.6)	<0.001	15 (23.8)	93 (30.9)	0.291
Cough	46 (7.3)	32 (8.8)	14 (5.2)	0.091	7 (11.1)	25 (8.3)	0.466
Admission temperature > 38°C^ 3^	124 (19.7)	108 (29.9)	16 (6.0)	<0.001	17 (27.0)	91/298 (30.5)	0.683
Admission pulse > 90/min ^4^	242 (38.5)	151 (41.8)	91 (34.1)	0.006	32 (51.6)	119/299 (39.8)	0.112
Admission systolic blood pressure < 90 mmHg ^4^	13 (2.1)	11 (3.1)	2 (0.8)	0.084	6 (10.0)	5 (1.7)	0.004
Dehydration	321 (50.7)	215 (59.1)	106 (39.4)	<0.001	45 (71.4)	170 (56.5)	0.034
Abdominal tenderness	330 (52.1)	199 (54.7)	131 (48.7)	0.148	29 (46.0)	170 (56.5)	0.164
Mental impairment	34 (5.4)	27 (7.2)	7 (2.6)	0.007	12 (19.0)	15 (5.0)	0.001
Lecocytosis ^5^	92 (14.8)	66 (18.2)	26 (10.1)	0.008	15 (23.8)	51/300 (17.0)	0.274
Leucopenia ^6^	27 (4.4)	19 (5.2)	8 (3.1)	0.235	5 (7.9)	14 (4.7)	0.345
Anaemia ^7^	44 (7.1)	29 (8.0)	15 (5.9)	0.344	6 (9.5)	23 (7.7)	0.611
Elevated urea ^8^	189 (30.6)	113 (31.4)	76 (29.6)	0.658	39 (61.9)	74 (24.9)	< 0.001

Data are number (%), except where stated.

^1^ Comparison of patients with blood culture taken and patients without blood culture taken.

^2^ Comparison of patients with a positive blood culture and patients with a negative blood culture among the 354 patients who had a blood culture taken.

^3^ n = 628.

^4^ n = 605.

^5^ White blood cell count >12.0 × 10^9^/L; n = 620.

^6^ White blood cell count <4.0 × 10^9^/L; n = 620.

^7^ Haemoglobin <11 g/dl in females and <13 g/dl in males; n = 619.

^8^ Urea >7.5 mmol/l; n = 617.

**Figure 2:**
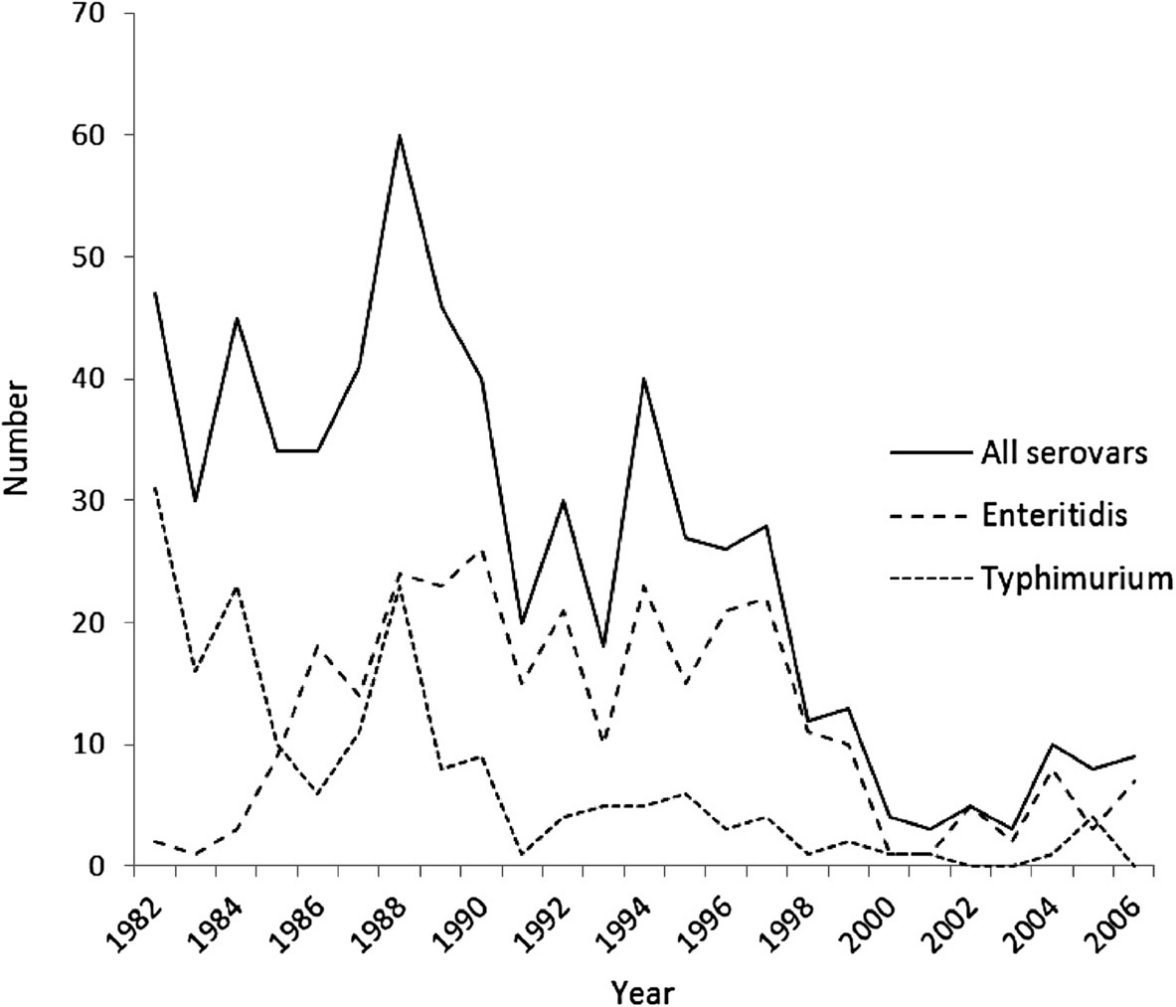
**Overall number of cases of non-typhoidal *Salmonella *gastroenteritis admitted to a hospital in Liverpool, UK between 1982 and 2006 inclusive by year and the number of cases due to serovar Typhimurium and serovar Enteritidis.**

Enteritidis and Typhimurium were the commonest serovars isolated from stool and blood although Virchow and Panama were more commonly associated with bacteraemia (Table 2). Among the serovar Enteritidis isolates, 160/294 (54%) were phage type 4 (PT4) and for Typhimurium 27/174 (15%) were phage type DT104. In the early 1980s Typhimurium was the dominant serovar, but was replaced by Enteritidis by the end of the decade (Figure 2). Total cases peaked in 1988, accounted for by high numbers of Enteritidis and Typhimurium. Enteritidis remained the dominant serovar during the 1990s. The total number of *Salmonella* cases declined from 1998 onwards. Each year cases peaked in the summer months.

**Table 2:** **Serovar distribution of *Salmonella *in blood and faeces for 633 adults**

**Serovar**	**Number of isolates from faeces (%)**	**Number of isolates from faeces in patients with blood culture done (%)**	**Number of isolates from blood (%)**	**Percentage of patients with faecal isolate also bacteraemic when blood culture done**
Total samples	633 (100)	364 (100)	63 (100)	17.3
Enteritidis	295 (46.6)	183 (50.3)	30 (47.6)	16.4
Typhimurium	175 (27.6)	96 (15.2)	9 (14.3)	9.4
Virchow	31 (4.9)	23 (6.3)	7 (11.1)	30.4
Panama	11 (1.7)	5 (1.4)	4 (6.3)	80.0
Kedougou	8 (1.3)	3 (0.8)	0 (0)	0
Hadar	7 (1.1)	3 (0.8)	1 (1.6)	33.0
Stanley	6 (1.0)	2 (0.6)	0 (0)	0
Newport	5 (0.8)	4 (0.8)	2 (3.2)	50.0
Anatum	5 (0.8)	2 (0.5)	0 (0)	0
Agona	4 (0.6)	2 (0.5)	0 (0)	0
Heidelberg	4 (0.6)	2 (0.5)	2 (3.2)	100
Infantis	4 (0.6)	0 (0)	0 (0)	0
Montevideo	4 (0.6)	2 (0.5)	0 (0)	0
Braenderup	3 (0.5)	1 (0.3)	1 (1.6)	100
Mbandka	1 (0.2)	1 (0.3)	1 (1.6)	100
New Brunswick	1 (0.2)	1 (0.3)	1 (1.6)	100
Stanleyville	1 (0.2)	1 (0.3)	1 (1.6)	100
Untyped	50 (7.9)	27 (7.4)	4 (6.3)	14.8
Other	18 (2.8)	6 (1.6)	0 (0)	0

Blood cultures were taken from 364 patients and they were significantly more likely to have had <4 days of symptoms before admission, a history of vomiting, fever, rigors, and headache on admission and pyrexia, tachycardia, dehydration and mental impairment on examination, and leuocytosis (Table 1). NTS was cultured from the blood of 63 patients, 10.0% of total, and 17.3% of those with blood cultures taken. Bacteraemic patients were significantly more likely to be ≥ 65 years old, with an underlying chronic disease including hypochlorhydria, to have a low systolic blood pressure with dehydration, mental impairment, and an elevated urea (Table 1). There was a significant increase in the proportion of patients with bacteraemia over time (31/222 (14.0%) during 1982–1991 compared with 32/142 (22.5%) during 1992–2006 (p = 0.046). In adults under 65 years the proportion of positive blood cultures was 12/63 (19.0%) in those with, and 24/225 (10.7%) in those without underlying chronic disease. For those 65 years or older the corresponding proportions were 17/50 (34.0%) in those with underlying disease and 10/26 (38.5%) in those without (Table 3). In a multivariable analysis, a positive blood culture was independently associated with an elevated urea (OR 3.38; 95% CI 2.30–7.68, p < 0.001) and mental impairment (OR 3.07, 95% CI 1.27–7.43. p = 0.013). All tested isolates were susceptible to ciprofloxacin by prevailing guidelines but ciprofloxacin MIC testing or nalidixic acid susceptibility was not performed.

**Table 3:** **Comparison by age group of the outcome in 633 adults admitted to hospital with non-typhoidal *Salmonella *gastroenteritis in Liverpool between 1982 and 2006 inclusive**

	**All patients**	**Age < 65 years with no underlying chronic disease**	**Age < 65 years with underlying chronic disease**	**p**^**1**^	**Age ≥ 65 years with no underlying chronic disease**	**Age ≥ 65 years with underlying chronic disease**	**P**^**2**^
Number	633	381	102		50	100	
Bacteremia	63/364 (17.3)	24/225 (10.7)	12/63 (19.0)	0.086	10/26 (38.5)	17/50 (34.0)	0.802
Given intravenous fluids	367 (58.0)	206 (54.1)	63 (61.8)	0.790	34 (68.0)	64 (64.0)	0.717
Given antibiotic in hospital	286 (45.2)	155 (40.7)	45 (44.1)	0.572	25 (50.0)	61 (61.0)	0.223
All complications	148 (23.4)	54 (14.2)	22 (21.6)	0.095	22 (44.0)	50 (50.0)	0.603
Specific complications ^3^							
Duration of admission > 21 days	66 (10.4)	14 (3.7)	11 (10.8)	0.009	12 (24.5)	30 (30.0)	0.563
Gastrointestinal complication ^4^	44 (7.0)	21 (5.5)	10 (9.8)	0.169	1 (2.0)	12 (12.0)	0.061
Renal impairment	26 (4.1)	4 (1.0)	6 (5.9)	0.008	8 (16.0)	8 (8.0)	0.163
Urinary tract infection	15 (2.4)	10 (2.6)	0 (0)	0.130	0 (0)	5 (5.0)	0.170
Pneumonia	14 (2.2)	2 (0.5)	2 (2.0)	0.198	3 (6.0)	7(7.0)	1.00
Hypotension	13 (2.1)	3 (1.0)	4 (4.0)	0.039	2 (4.0)	4 (4.0)	1.00
Reactive arthritis/uveitis	9 (1.4)	8 (2.1)	1 (1.0)	0.692	0 (0)	0 (0)	-
Central nervous complication^ 5^	5 (0.8)	0 (0)	2 (2.0)	0.044	2 (4.0)	1 (1.0)	0.258
Vascular complication^ 6^	4 (0.6)	0 (0)	0 (0)	-	1 (2.0)	3 (3.0)	1.00
Total deaths	14 (2.2)	0 (0)	1 (1.0)	0.211	1 (2.0)	12 (12.0)	0.061
Attributable deaths	9 (1.4)	0 (0)	1(1.0)	0.211	1 (2.0)	7 (7.0)	0.270

Data are number (%), except where stated.

^1^ Univariate statistical comparison of those age < 65 with no underlying disease versus age < 65 with underlying disease.

^2^ Univariate statistical comparison of those age ≥ 65 with no underlying disease versus age ≥ 65 with underlying disease.

^3^ 14 patients with site specific complications had more than one site affected.

^4^ Ileus/subacute obstruction (7); severe colitis (7); prolonged diarrhoea (diarrhoea persisting for > two weeks) (6); irritable bowel syndrome (5); *Clostridium difficile* colitis (3); appendicitis (2); recurrent diarrhoea (recurrence of diarrhoea after intial recovery) (2); toxic colonic dilatation (2); erosive gastritis (1); exacerbation of pre-existing inflammatory bowel disease (1); gastrointestinal bleeding (1); persistent vomiting (1).

^5^ Acute confusion (3), coma with possible encephalopathy (2).

^6^ Cerebrovascular accident (2); pulmonary embolus, gangrene and cerebrovascular accident (1); septic thrombophlebitis (1).

Antibiotics were prescribed after hospital admission in 286 (45%) of the 633 patients and in 54 (86%) of the 63 patients with a positive blood culture. Ciprofloxacin was prescribed in 198 (69%) of the 286 cases where an antibiotic was prescribed and was first used in 1987. Between 1987 and 1996 it was used in 157/188 (84%) of cases and between 1997 and 2006 it was used in 41/58 (71%) of cases. Other antibiotics commonly prescribed were: an injectable second or third generation cephalosporin (n = 33); co-trimoxazole or trimethoprim (n = 31); and ampicillin (n = 23).

The main complications were gastrointestinal or prolonged hospital stay (Table 3). The proportion of patients with a complication did not change significantly over time (97/397 (24.4%) during 1982–1991 compared with 51/236 (21.6%) during 1992–2006 (p = 0.439). Vascular complications only occurred in those ≥ 65 years old, and no patients had a confirmed endovascular infection or endocarditis. Of the 13 patients with both bacteraemia and underlying vascular or valvular heart disease, all but one aged ≥50 years, only one developed a diagnosed vascular complication. This patient had a pulmonary embolus, peripheral gangrene and a cerebrovascular accident and subsequently died. A further four of these 13 patients developed acute renal failure secondary to shock (n = 1) or dehydration (n = 3) and it is possible they had an undiagnosed endovascular lesion. Three of these 13 patients were treated with ciprofloxacin, but these did not include the patients who developed complications. No patient developed osteomyelitis although reactive arthritis and/or uveitis occurred in nine (1.4%) patients, all aged below 50 years. A *Salmonella* urinary infection was present in 15 (2.4%) of all patients and 15/466 (3.2%) patients who had a urine cultured. There were no known urinary tract anatomical abnormalities in these patients. Of patients who had both blood and urine cultured 5/12 (41.7%) patients with a *Salmonella* bacteruria were also bactaeremic compared with 48/290 (16.6%) who were not bacteruric (p = 0.041).

Fourteen patients were known to have died within 6 months of admission. All deaths were in patients older than 55 years and rose with increasing age (Figure 1). The youngest was a man of 57 years with alcoholic liver disease. *Salmonella* made a significant contribution to death in nine of the patients who died in hospital within 30 days of admission giving an attributable 30 day case fatality rate of 1.5%.

The proportion of patients with complicated or fatal disease in the group of patients who had a blood culture taken was 91/364 (25.0%) and was not significantly different from 57/269 (21.2%) in those patients who were not cultured (p = 0.269). The type of complications did not significantly vary between the two groups. In the patients who had a blood culture taken, complicated or fatal disease was significantly more common in the elderly, those with chronic disease, hypochlorhydria, duration of symptoms > 4 days prior to admission, the absence of a history of fever, the presence of symptoms of dehydration and new onset confusion or coma at the time of admission, leucocytosis, elevated urea and a positive blood culture for salmonella (Table 4). In a multivariable model, bacteraemia; new onset confusion or coma; duration of symptoms > 4 days prior to admission; dehydration; and the absence of a history of fever remained significant. There were five (11.9%) deaths in the 42 patients aged 50 years or more with bacteraemia but none in the 21 bactaeremic patients aged below 50 years.

**Table 4:** **Univariate clinical and demographic associations with complicated or fatal disease in 364 adults admitted to hospital with non-typhoidal *Salmonella *gastroenteritis who had a blood culture taken**

	**Complicated or fatal disease**	**Uncomplicated disease**	**OR (95% CI)**	**p**^**1**^	**Adjusted OR (95% CI)**	**p**^**2**^
Number (%)	91 (25.0)	273 (75.0)				
Age ≥ 65 years	35 (38.5)	41 (15.0)	3.54 (2.07–6.05)	<0.001		
Male gender	37 (40.7)	134 (49.1)	0.71 (0.44–1.15)	0.183		
Current smoker	30 (33.0)	83 (30.4)	1.13 (0.66–1.93)	0.110		
Current alcohol user	48 (52.7)	176 (64.5)	0.06 (0.37–1.02)	0.062		
Recent travel history	14 (15.4)	35 (12.8)	1.24 (0.63–2.42)	0.595		
Chronic disease	41 (45.1)	72 (26.4)	2.29 (1.40–3.75)	0.002		
Hypochlorhydria	15 (16.5)	24 (8.8)	2.05 (1.02–4.10)	0.050		
Recent antibiotic therapy	7 (7.7)	25 (9.2)	0.83 (0.35–1.98)	0.831		
Duration symptoms > 4 days	47 (51.6)	87 (31.9)	2.28 (1.41–3.70)	0.001	2.48 (1.44–4.27)	0.001
Bloody diarrhoea	14 (15.4)	51 (18.7)	0.79 (0.42–1.51)	0.530		
Abdominal pain	69 (75.8)	215 (78.8)	0.85 (0.48–1.48)	0.561		
Vomiting	65 (71.4)	200 (73.3)	0.91 (0.54–1.55)	0.786		
Pyrexia	35 (38.5)	145 (53.1)	0.55 (0.34–0.90)	0.016	0.56 (0.32–0.95)	0.033
Rigors	14 (15.4)	69 (23.5)	0.54 (0.29–1.01)	0.060		
Headache	20 (22.0)	88 (32.2)	0.59 (0.34–1.03)	0.065		
Cough	11 (12.1)	21 (7.7)	1.65 (0.76–3.57)	0.204		
Admission temperature > 38°C ^3^	24 (26.4)	84 (31.1)	0.81 (0.46–1.42)	0.508		
Admission pulse > 90/min ^4^	46 (50.5)	105 (38.9)	1.61(1.00–2.59)	0.065		
Dehydration	67 (73.6)	148 (54.2)	2.36 (1.40–3.98)	0.001	1.90 (1.07–3.38)	0.029
Abdominal tenderness	44 (48.4)	155 (56.8)	0.71 (0.44–1.15)	0.182		
Mental impairment	18 (19.8)	9 (3.3)	7.23 (3.12–16.77)	<0.001	4.80 (1.91–12.07)	0.001
Leococytosis ^5^	25 (27.5)	41 (15.1)	2.13 (1.21–3.77)	0.012		
Leucopenia ^6^	4 (4.4)	15 (5.5)	0.79 (0.26–2.44)	0.792		
Anaemia ^7^	9 (9.9)	20 (7.4)	1.38 (0.61–3.16)	0.503		
Elevated urea ^8^	46 (50.5)	67 (24.9)	3.08 (1.88–5.06)	< 0.001		
Serovar Enteritidis infection	47 (51.6)	136 (49.8)	1.08 (0.65–1.78)	0.856		
Serovar Typhimurium infection	18 (19.8)	78 (28.6)	0.62 (0.33–1.14)	0.131		
Serovar Virchow infection	7 (7.7)	16 (5.9)	1.34 (0.48–3.61)	0.709		
Positive blood culture	37 (40.7)	26 (9.5)	6.51 (3.64–11.64)	<0.001	5.34 (2.86–9.95)	0.001

Data are number (%), except where stated.

^1^ Univariable comparison of patients with complicated or fatal disease compared with those with uncomplicated disease.

^2^ Adjusted comparison from multivariable logistic regression.

^3^ n = 361.

^4^ n = 354.

^5^ White blood cell count >12.0 × 10^9^/L; n = 363.

^6^ White blood cell count <4.0 × 10^9^/L; n = 363.

^7^ Hemoglobin <11 g/dl in females and <13 g/dl in males; n = 363.

^8^ Urea >7.5 mmol/l; n = 360.

## Discussion

In this large cohort of adults admitted with community acquired NTS gastroenteritis to a single hospital centre over a 25-year period, secondary bacteraemia occurred in 17.3% of patients cultured and 10.0% overall. These secondary bacteraemia figures are higher than the 6.7%, 7% and 3.8% reported in other adult studies [8-10] and 6.5% to 11.8% in children [6,7,12,14]. Only two of these studies performed blood cultures on every patient [7,10]. In the current study secondary bacteraemia occurred in one in ten healthy adults aged <65 years who had a culture taken, consistent with the data from healthy children, but almost one in five of those with underlying disease. In elderly patients (≥ 65 years) one third had bacteraemia and, unlike the younger adults, this proportion did not vary significantly between those with, and without, chronic underlying disease. The reason for the higher level of secondary bacteraemia is difficult to determine in this retrospective study. Patients may have been more likely to have had blood cultured as many were admitted to the infectious diseases ward where this is routine. The contribution of antibiotic resistance cannot be determined because of the lack of susceptibility data on the isolates.

This study suggests that secondary bacteraemia, new onset confusion or coma, a prolonged duration of symptoms before admission, dehydration and the absence of a history of fever are significantly associated with a complicated or fatal disease in non-HIV infected adults admitted to hospital with NTS gastroenteritis. Age and underlying chronic disease did not remain in the final model and suggest that bacteraemia, prolonged disease and dehydration are the critical factors leading to a complicated outcome. Clinical features such as rigors, fever, tachycardia, or leucocytosis did not distinguish bacteraemic from non-bacteraemic patients. Although an association of fever or prolonged fever has been found with bacteraemia in children [7,12], a study of 105 adults, found that the presence of fever or neutrophilia was not significantly associated with bacteraemia [9]. Bacteraemia was more common in infections with serovar Virchow and Panama, as recognized in other studies [10,17,18].

Complicated disease developed in almost one quarter of patients, with gastrointestinal complications and a prolonged hospital stay reported most frequently. The range of complications was consistent with other studies except for a notable lack of endovascular infection [2,19]. Previous studies have reported endovascular infections occurring in between 7 and 41% of cases of NTS bacteraemia in adults aged >50 years [11,20-22]. Our study found only one diagnosed vascular complication among 42 bacteraemic patients aged >50 years. It is possible that the risk may be less than has previously been reported, that less invasive serovars are seen at this centre or that this complication was simply under-diagnosed as routine CT scanning was not performed. The occurrence of reactive arthritis or uveitis in 1.4% of patients, all younger than 50 years, was consistent with previous studies [23] but the 2.4% of patients with a urinary infection was higher than expected [19,24]. The absence of renal tract abnormalities or immunosuppression and the finding that 42% of bacteriuric patients were bacteraemic suggests infection secondary to bacteraemia rather than an ascending urinary infection [24]. Almost all the deaths were in patients aged 65 years or more [8,9]. Studies that report deaths in younger patients tend to be from earlier studies or include primary extra-intestinal infections [5,25,26]. Although more patients with severe illness and complicated outcomes received antibiotics after admission, the retrospective nature of this study does not allow examination of their effectiveness. It is notable, however, that almost one third of patients over 65 years were not given an antibiotic after admission to hospital, and this even included some patients who had a complicated course, and suggests that the need for antibiotics in this age group is insufficiently recognised.

This study was limited by being retrospective. Data were collected on a standardised case report form over the whole of the twenty-five year period, and where possible, the inaccuracies associated with retrospective data have been minimised. The study was restricted to hospital-admitted and bacteriologically-proven cases. A further important limitation was that not all patients had a blood culture taken and that those cultured were sicker than those who were not. We excluded patients known to be HIV infected but cannot be completely certain that some were not missed as HIV testing was not routinely performed in this patient group over the course of the study. During this period most HIV positive adults in the Liverpool area requiring hospital admission were admitted to the same hospital unit, and the prevalence of HIV in the Liverpool area was low. We cannot be sure that bacteraemia isolates were susceptible to ciprofloxacin because none had an MIC performed or testing for nalidixic acid susceptibility. Nalidixic acid resistant isolates with decreased ciprofloxacin susceptibility have been associated with a three-fold increased risk of invasive disease and death in adults in Denmark [27].

The study is representative of nationally reported trends in NTS infections in the last 25 years. Peaks of cases in the 1980s and 1990s coincided with the epidemic in serovar Enteritidis PT4 cases linked to poultry and eggs. The decline in the last decade has been attributed to better animal husbandry, vaccination of chicken flocks and improved kitchen hygiene [28].

## Conclusions

In conclusion, this study suggests that secondary bacteraemia is associated with an adverse outcome in non-HIV infected adults admitted to hospital with NTS gastroenteritis. Blood cultures should be performed in adults ill enough to be admitted to hospital with gastroenteritis. Antibiotic therapy, in addition to careful rehydration, should be considered in patients at high risk of secondary bacteraemia, including elderly patients (>65 years) and younger adults with relevant underlying chronic conditions [29,30].

## Competing interests

The authors have no conflicts of interest to declare.

## Authors’ contributions

CMP, SJR, ADH and NJB designed the study. All authors participated in data collection. CMP analysed the data and wrote the first draft. All authors revised the manuscript for important intellectual content and read and approved the final version.

## Pre-publication history

The pre-publication history for this paper can be accessed here:

http://www.biomedcentral.com/1471-2334/13/107/prepub
